# Adaptive changes in striatal projection neurons explain the long duration response and the emergence of dyskinesias in patients with Parkinson’s disease

**DOI:** 10.1007/s00702-022-02510-8

**Published:** 2022-05-10

**Authors:** Björn Falkenburger, Theodoros Kalliakoudas, Heinz Reichmann

**Affiliations:** 1 Department of Neurology, TU Dresden, Fetscherstraße 74, 01307 Dresden, Germany; 2 Deutsches Zentrum für Neurodegenerative Erkrankungen (DZNE), Dresden, Germany

**Keywords:** Parkinson’s disease, Striatum, Medium spiny neurons, Spiny projection neurons, Homeostatic plasticity, Dyskinesias

## Abstract

Neuronal activity in the brain is tightly regulated. During operation in real time, for instance, feedback and feedforward loops limit excessive excitation. In addition, cell autonomous processes ensure that neurons’ average activity is restored to a setpoint in response to chronic perturbations. These processes are summarized as homeostatic plasticity (Turrigiano in Cold Spring Harb Perspect Biol 4:a005736–a005736, 2012). In the basal ganglia, information is mainly transmitted through disinhibition, which already constraints the possible range of neuronal activity. When this tightly adjusted system is challenged by the chronic decline in dopaminergic neurotransmission in Parkinson’s disease (PD), homeostatic plasticity aims to compensate for this perturbation. We here summarize recent experimental work from animals demonstrating that striatal projection neurons adapt excitability and morphology in response to chronic dopamine depletion and substitution. We relate these cellular processes to clinical observations in patients with PD that cannot be explained by the classical model of basal ganglia function. These include the long duration response to dopaminergic medication that takes weeks to develop and days to wear off. Moreover, dyskinesias are considered signs of excessive dopaminergic neurotransmission in Parkinson’s disease, but they are typically more severe on the body side that is more strongly affected by dopamine depletion. We hypothesize that these clinical observations can be explained by homeostatic plasticity in the basal ganglia, suggesting that plastic changes in response to chronic dopamine depletion and substitution need to be incorporated into models of basal ganglia function. In addition, better understanding the molecular mechanism of homeostatic plasticity might offer new treatment options to avoid motor complications in patients with PD.

## Introduction

Neuronal activity in the brain is tightly regulated. During operation in real time, for instance, feedback and feedforward loops limit excessive excitation. In addition, cell autonomous processes ensure that neurons’ average activity is restored to a setpoint in response to chronic perturbations. These processes are summarized as homeostatic plasticity (Turrigiano 2012). In the basal ganglia, information is mainly transmitted through disinhibition, which already constraints the possible range of neuronal activity. When this tightly adjusted system is challenged by the chronic decline in dopaminergic neurotransmission in Parkinson’s disease (PD), homeostatic plasticity aims to compensate for this perturbation. We here summarize recent experimental work from animals demonstrating that striatal projection neurons adapt excitability and morphology in response to chronic dopamine depletion and substitution. We relate these cellular processes to clinical observations in patients with PD that cannot be explained by the classical model of basal ganglia function. These include the long duration response to dopaminergic medication that takes weeks to develop and days to wear off. Moreover, dyskinesias are considered signs of excessive dopaminergic neurotransmission in Parkinson’s disease, but they are typically more severe on the body side that is more strongly affected by dopamine depletion. We hypothesize that these clinical observations can be explained by homeostatic plasticity in the basal ganglia, suggesting that plastic changes in response to chronic dopamine depletion and substitution need to be incorporated into models of basal ganglia function. In addition, better understanding the molecular mechanism of homeostatic plasticity might offer new treatment options to avoid motor complications in patients with PD.

### Dopamine depletion in Parkinson’s disease and basal ganglia models

Parkinson’s disease (PD) is the second most common neurodegenerative disease. Its motor symptoms are caused by degeneration of dopaminergic neurons of the substantia nigra pars compacta (SNc) and deficient dopaminergic neurotransmission in the striatum. Accordingly, the classical motor symptoms of PD are alleviated by dopaminergic medications (Obeso et al. 2017). Patients with PD also show axial and non-motor symptoms, which for the most part do not respond to dopaminergic medications. Intraneuronal aggregates of the protein α-synuclein can be found throughout the brain and the peripheral nervous system and likely underlie the axial and non-motor symptoms. Discovering dopamine deficiency in PD and developing dopaminergic treatments was one of the great medical achievements of the twentieth century. Yet, basic aspects of basal ganglia physiology have remained obscure, and the long-term effects of dopaminergic treatments are not satisfactory (Obeso 2019).

**Fig. 1:**
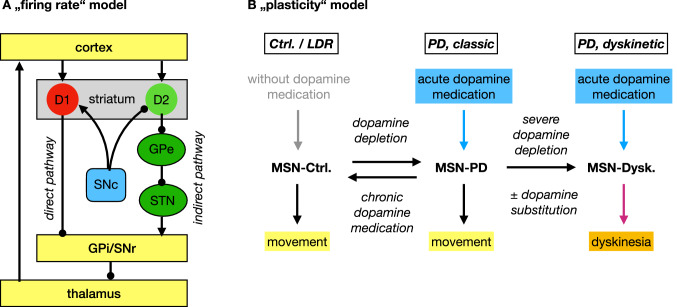
Model of basal ganglia function. **A** Classical model explaining firing rate changes in basal ganglia nuclei during dopamine depletion. **B** Summary of changes over time in Parkinson’s disease (PD): In control conditions, movement can be initiated without dopaminergic medication. In early PD, acute administration of dopaminergic medication facilitates movement (short duration response, SDR). Chronic dopaminergic medication allows movement even without dopaminergic medication (long duration response, LDR). In advanced PD, with severe dopamine depletion, dopaminergic medication elicits dyskinesias. Differences between MSN-Ctrl., MSN-PD and MSN-Dysk. are summarized in Table 1

The classical model of basal ganglia function by Albin and de Long (Fig. 1A) is incomplete in many aspects, but still helpful to outline the overall architecture (Albin et al. 1989). The striatum is the largest structure of the basal ganglia and the strong cortico-striatal projection considered its main input. The SNc also projects mainly to the striatum. Hence, the striatum constitutes the brain region in which dopamine deficiency results in PD motor symptoms. Spiny projection neurons, traditionally termed medium spiny neurons (MSN), make up the majority of striatal neurons. MSN carry either D1 or D2 dopamine receptors. D1-MSN project directly to the internal segment of the pallidum (GPi) and further basal ganglia output structures (direct pathway), whereas D2-MSN project to the basal ganglia output structures as part of the indirect pathway. D1 dopamine receptors are excitatory and D2 receptors are inhibitory. As predicted by the classical model, D1-MSN are hypoactive and D2-MSN are hyperactive when recorded in mice with chronic dopamine depletion using optogenetics (Parker et al. 2018).

Adaptations over time are not represented in the classical model. More recent basal ganglia models use oscillations to explain the emergence of tremor and the effects of deep brain stimulation (Hutchison et al. 2004). Yet, they also do not contain a representation of changes over time. In the following, we illustrate that these long-term changes during dopamine depletion and with dopaminergic medications are important for PD pathophysiology.

### The long duration response (LDR)

Acute administration of levodopa or dopamine receptor agonists alleviates PD motor symptoms and increases, for instance, the speed of finger tapping in patients with PD (Nutt et al. 1997). The effect of a single dose is entirely reversible after 24 h and therefore termed the short duration response (SDR). Patients that chronically receive levodopa show an additional long duration response (LDR) that takes weeks to build up and at least days to disappear. The LDR is superimposed with the SDR and cannot be explained by pharmacokinetics. Different explanations for the LDR have been provided, including storage of levodopa. Yet, the LDR is also observed with short acting dopamine receptor agonists (Stocchi et al. 2001).

Based on the available data, we currently assume that the SDR results from acute changes in basal ganglia firing rates as represented in the Albin and de Long model (Fig. 1A). The LDR, in contrast, results from plastic changes in neuronal excitability and connectivity (Fig. 1B). The LDR was also observed in the ELLDOPA study where patients were treated with placebo or levodopa up to 600 mg/d for 1 year. Patients on 600-mg levodopa showed an additional increase in motor performance after a stable dose of levodopa was reached, and much better motor performance at the end of the study after levodopa had been withdrawn for 2 weeks (Fahn et al. 2004). In a cohort of initially drug naïve patients with advanced PD, the LDR was recently estimated by comparing motor performance after 1 or 2 years of levodopa treatment and overnight withdrawal to baseline values (Cilia et al. 2020). In all of these studies, the size of the LDR was substantially larger than the SDR, highlighting the therapeutic potential of understanding the cellular mechanism that underlies the LDR.

Functionally, the LDR stores the effects of dopaminergic medications, acting like a buffer and contributing to the fact that motor performance generally does not fluctuate during the honeymoon period of PD, even if dopaminergic medication is taken on only three timepoints per day. At this stage, motor performance generally does not change when a patient forgets to take a medication. As a consequence, patients can be under the false impression that their medication is ineffective. When clinicians want to verify that motor symptoms indeed respond to dopaminergic medication in these patients, they need to plan for a longer time of dopamine withdrawal than typically used in fluctuating patients. In this context we note that the acute levodopa challenge only assesses the SDR, whereas a chronic levodopa test also incorporates the LDR. Consequently, the latter has been found to be more sensitive.

### Motor fluctuations and dyskinesias

In patients with advanced PD, the effects of dopaminergic medication typically “wear off”. This can be regarded as a loss of the buffering effect of the LDR. In addition, dopaminergic medication can induce excessive involuntary movements termed dyskinesias. Dyskinesias usually occur at the peak of the drug’s serum concentration (“peak dose dyskinesia”), suggesting that they represent an overshoot of the therapeutic effect (Espay et al. 2018). Yet, several facts indicate that this view is too simplistic and that dyskinesias result from plastic changes in neuronal excitability and connectivity. First, dyskinesias occur in advanced PD but not in healthy individuals or early PD patients. If dyskinesias would simply result from excessive dopaminergic neurotransmission, they should be more prominent in patients with less dopamine depletion. Similarly, dyskinesias are generally stronger on the side of the body with more severe PD symptoms. Second, “diphasic” dyskinesias can occur at the onset and/or offset of the dopaminergic effect, i.e., with low serum concentrations of the dopaminergic drug and not at the peak. The ability to produce dyskinesias therefore requires advanced degeneration of dopaminergic axon terminals and results from plastic changes to this degeneration. We refer to this process as “dyskinesia priming”. In addition, the expression of dyskinesias requires the administration of a dopaminergic medication. We refer to this process as “dyskinesia triggering”.

Taken together, motor fluctuations are characterized by three processes, the loss of the LDR, dyskinesia priming, and dyskinesia triggering. It is conceivable that different cellular and molecular events underlie these processes. Motor fluctuations are addressed therapeutically by reducing fluctuations in the serum concentration of dopaminergic drugs, e.g., by more frequent administration, by blocking the dopamine degrading enzymes COMT and MAO, and by medication pumps (Obeso et al. 2017; Espay et al. 2018). This principle certainly prevents dyskinesia triggering, but we do not know whether it also affects the LDR and dyskinesia priming. We generally assume that this is the case. Indeed, continuous administration of the dopamine receptor agonist rotigotine was able to reduce dyskinesias (Mouradian et al. 1990; Stockwell et al. 2010).

In rodents, dopaminergic drugs can induce abnormal involuntary movements (AIM), which are a model for dyskinesias. Similar to dyskinesias of non-human primates, rodent AIM require chronic dopamine depletion to occur (Cenci 2014). In addition, AIM are typically induced by repeated administration of levodopa, hence the term “levodopa-induced dyskinesias (LID)”. Both factors support the notion that they result from plastic changes in neuronal excitability and connectivity. AIM were reduced in a rat model by transducing inhibitory D2 receptors into serotoninergic axon terminals (Sellnow et al. 2019), suggesting that uptake and activation of levodopa by serotoninergic axon terminals contributes to dyskinesia triggering. Yet, this process cannot account for all aspects of dyskinesia priming.

“Tardive” dyskinesias are observed in patients after administration of dopamine receptor antagonists, suggesting that they result from similar adaptation processes as dyskinesias in patients with PD, with physiological dopaminergic neurotransmission taking the role of levodopa treatment (Blanchet and Lévesque 2020; Ali et al. 2020). Accordingly, tardive dyskinesias can be alleviated by dopamine receptor antagonists.

### Adaptations in D1-MSN and D2-MSN

Most evidence about the functioning of the basal ganglia circuitry and plastic changes with dopamine depletion and dopaminergic medications were obtained in animal models. In mice, MSN show homeostatic changes of excitability with dopamine depletion and substitution (Azdad et al. 2009; Fieblinger et al. 2014; Parker et al. 2018). D1-MSN are excited by dopamine and become hypoactive with dopamine depletion. Yet, they increase excitability with dopamine depletion—as if to compensate for the lacking dopamine effect. Levodopa substitution reverts excitability in D1-MSN. D2-MSN, in contrast, are inhibited by dopamine and hyperactive with dopamine depletion. D2-MSN reduce their excitability with dopamine depletion and levodopa reverts excitability. These changes in excitability are accompanied by changes in MSN morphology (complexity of the dendritic arborization) and spine density. Interestingly, however, both MSN types show a decreased spine density and less complex dendritic arborizations with dopamine depletion (Fieblinger et al. 2014; Suarez et al. 2014, 2016, 2018; Gagnon et al. 2017), and the same changes in MSN morphology are also observed in MPTP-treated monkeys (Villalba et al. 2009) and in the brains of patients with PD (Zaja-Milatovic et al. 2005). The importance of dopamine for these morphological adaptations in MSN is highlighted by experiments in cultured primary MSN where dopamine substitution increased number of spines (Fasano et al. 2013) and by optogenetic experiments in mice where dopaminergic stimulation within a defined time window promoted spine enlargement after dopaminergic stimulation (Yagishita et al. 2014). Furthermore, the extent of spine loss changes with dopamine concentration in Aphakia mice (Alberquilla et al. 2020) and depends on dopamine receptors (Suarez et al. 2020). Findings from animal studies are summarized in Table 1.

**Table 1:** Changes in rodent MSN observed with dopamine depletion and substitution

	Ctrl	MSN-PD	MSN-Dysk
Excitability	Normal	D1: increased^a^	D1: partially restored^b^
		D2: decreased^c^	D2: partially restored^b^
Spine density	Normal	D1: decreased^d^	D1: decreased^d^
		D2: decreased^d^	D2: normalized^d^
Dendritic arbor	Normal	D1: decreased^e^	potentially restored^f^
		D2: decreased^e^	

^a^Azdad et al. (2009); Fieblinger et al. (2014); Suarez et al. (2016, 2018); Alberquilla et al. (2020)

^b^Fieblinger et al. (2014;) Suarez et al. (2016, 2018)

^c^Fieblinger et al. (2014;) Suarez et al. (2016) but note Suarez et al. (2018); Alberquilla et al. (2020)

^d^Villalba et al. (Suarez et al. (2016); Gagnon et al. (2017; 2009) but note Fieblinger et al. (2014)

^e^Fieblinger et al. (2014); Suarez et al. (2018), but unaltered in Suarez et al. (2014, 2016)

^f^Fieblinger et al. (2018); Witzig et al. (2020)

Long-term potentiation (LTP) is the most studied form of neuronal adaptation. Striatal LTP is reduced in mice with dopamine depletion and restored by levodopa administration (Picconi et al. 2003, 2018; Calabresi et al. 2015; Schirinzi et al. 2016). After induction of AIM, mice show impaired depotentiation by low frequency stimulation (Picconi et al. 2003) and reduced long term depression. LTP is associated with spine growth. Reduced LTP with dopamine depletion is therefore consistent with the reduced spine density observed in PD patients and animal models. In contrast, it is hard to reconcile the dependence of LTP and dyskinesia on D1 receptors (Calabresi et al. 2000) with the fact that levodopa reverses spine pruning in D2-MSN but not in D1-MSN (Suarez et al. 2018). These discrepancies between excitability, LTP and spine density indicate that adaptations differ between D1-MSN and D2-MSN and between excitability, synaptic plasticity and morphological changes.

D1-MSN appear more important for the development of dyskinesias in PD models. For instance, only D1-MSN show a pronounced and dose dependent transcriptional response to levodopa treatment after dopamine depletion (Heiman et al. 2014). Moreover, signaling downstream of D1 receptors correlates linearly with dyskinesia severity (Aubert et al. 2005), internalizing or depleting D1 receptors reduces AIM (Darmopil et al. 2009; Ahmed et al. 2010), and activating D1-MSN is sufficient to induce AIM (Perez et al. 2017). Yet, D2 receptor agonists are just as well able to prime and trigger AIM and dyskinesias as D1 receptor agonists are (Huot et al. 2013). Moreover, high frequency stimulation of the subthalamic nucleus can elicit dyskinesias, even though the subthalamic nucleus is part of the indirect pathway as D2-MSN are. Finally, tardive dyskinesias are caused by D2 receptor antagonists such as haloperidol. Still, most dopamine agonists currently used to treat PD patients are selective for D2-type receptors (mainly D3) due to the more common psychiatric and cardiovascular side effects of D1 receptor agonists (Gerlach et al. 2003). Yet, a balanced activation of D1 and D2 receptors by levodopa seems to offer several advantages as compared to dopamine receptor agonists. In mice with dopamine depletion, only levodopa was able to restore the association of MSN activity with locomotion whereas receptor agonists were not (Parker et al. 2018).

Optogenetic recordings in freely moving mice demonstrated that both D1-MSN and D2-MSN are activated by movement (Cui et al. 2013; Parker et al. 2018). These observations are surprising in the light of the classical rate model where D1-MSN and D2-MSN generally respond oppositely. It is assumed that network effects underlie the similar response of D1-MSN and D2-MSN to movement, and it is conceivable that the similar regulation by movement might be related to the similar change in spine density. Indeed, there are also strong regional differences between MSN populations. Large-scale recordings in feely moving animals have demonstrated regional clusters of MSN that are responsible for specific tasks (Klaus et al. 2017) and fire together irrespective of whether they are D1-MSN or D2-MSN. Moreover, changes in neuronal activity rates during dopamine depletion or substitution differ strongly between individual neurons, average values are driven by outliers (Ryan et al. 2018) (Ryan et al., 2018). These findings indicate that the expression of D1 vs. D2 dopamine receptors might not be the most important property of striatal MSN. We have focused on MSN here, but note that striatal interneurons also play an important role for physiology and pathophysiology (Maurice et al. 2015; Tanimura et al. 2019).

### Conclusions and further directions

Taken together, dopamine depletion and dopaminergic drugs entail not only an acute response, but also plastic changes in the brain. We know that dopamine receptor antagonists can cause tardive dyskinesias and that dopamine depletion in PD is required for priming dyskinesias in these patients. However, we do not know whether initiating treatment early prevents dyskinesia priming or supports it, and whether medications differ in their capacity to do so.

Studying plastic changes in MSN morphology and excitability might be able to resolve the cellular and molecular processes underlying these long-term plastic changes in patients. Indeed, calcium channel antagonists were able to block both spine loss and the emergence of AIM in a mouse model of dopamine depletion (Steece-Collier et al. 2019). Moreover, the LDR to levodopa in the ELLDOPA study was more pronounced in the dominant hand, i.e., the one that is generally used more (Kang et al. 2012). This finding indicates that the LDR is modulated by exercise. Interestingly, spine loss at MSN after dopamine depletion can also be compensated by exercise (Petzinger et al. 2013), consistent with the hypothesis that the two processes are related.

Starting 4 years after clinical diagnosis of PD, virtually all dopaminergic axon terminals in the striatum are lost (Kordower et al. 2013). This raises the question why we have to increase medication in patients with PD beyond 4 years after diagnosis. In the context of the facts noted above, it is intriguing to speculate that dopaminergic medication is increased to compensate for the reduced LDR in advanced PD.

High frequency stimulation (HFS) of the subthalamic nucleus (STN) reduces PD motor symptoms to a similar extent as dopaminergic medication does. This raises the question whether STN-HFS, too, can elicit a LDR, or whether only dopaminergic medication can do that. One study showed worse motor performance in overnight medication withdrawal in patients with STN-HFS but without chronic dopaminergic medication (Wider et al. 2006), suggesting that STN-HFS does not produce its own LDR. Yet, patients with STN-HFS that do not require dopaminergic medication after surgery are usually tremor-dominant, suggesting that the LDR might be less pronounced for tremor than for akinetic-rigid symptoms. In our own experience, symptoms appear to respond less quickly to changes in DBS programming in advanced PD than observed directly after surgery, suggesting that STN-HFS can induce an LDR, consistent with the hypothesis that homeostatic plasticity underlies the LDR, which is a general mechanism by which the nervous system responds to perturbations. Long-lasting electrophysiological changes were observed during DBS surgeries (Luo et al. 2018), but this issue will require further investigation.
